# Tapering of Biological Agents in Juvenile ERA Patients in Daily Clinical Practice

**DOI:** 10.3389/fmed.2021.665170

**Published:** 2021-05-07

**Authors:** Chun-Hua Liao, Bor-Luen Chiang, Yao-Hsu Yang

**Affiliations:** ^1^Department of Pediatrics, National Taiwan University BioMedical Park Hospital, Hsin-Chu, Taiwan; ^2^Department of Pediatrics, National Taiwan University Hospital, Taipei, Taiwan; ^3^Department of Medical Research, National Taiwan University Hospital, Taipei, Taiwan; ^4^Department of Pediatrics, National Taiwan University Hospital, Hsin-Chu Branch, Hsin-Chu, Taiwan

**Keywords:** enthesitis-related arthritis, biologics, tapering, withdrawal, flare-up

## Abstract

**Objectives:** We aim to evaluate the proportion and characteristics of enthesitis-related arthritis (ERA) patients in whom medications can be withdrawn in daily practice and to analyze the factors associated with flare-ups during medication tapering of these patients.

**Methods:** We retrospectively reviewed records of patients under 16 years old diagnosed with ERA from April 2001 to March 2020 in one tertiary medical center in Taiwan. Patients were categorized by different medication uses: conventional disease modifying anti-rheumatic drugs (cDMARDs) only and cDMARDs plus biologics. Demographics, laboratory data, presence of uveitis, and medication withdrawal rate were analyzed. Subgroup analysis was performed in the patients with cDMARDs plus biologics to identify factors associated with flare-ups during medication tapering of these patients. Statistical analysis was performed using R (v3.6.0).

**Results:** There were 75 juvenile ERA patients with a median onset age of 10.28 years old. Nineteen (25.3%) patients used cDMARDs for disease control; 56 (74.7%) patients depended on cDMARDs plus biologics. Poly-articular involvement was noted in 29 (38.7%) patients, and it occurred more frequently in the cDMARDs plus biologics subgroup (cDMARDs only, 5.3%; cDMARDs plus biologics, 53.6%; *P* = 0.0001). ANA positivity was observed in 18 (24.0%) patients, and it occurred more frequently in the cDMARDs plus biologics subgroup (cDMARDs, 0%; cDMARDs plus biologics, 32.1%; *P* = 0.0038). The overall medication withdrawal rate was 34.7%, and it occurred more frequently in patients with cDMARDs only (cDMARDs only, 84.2%; cDMARDs plus biologics, 17.9%; *P* < 0.001). In the subgroup analysis of patients with cDMARDs plus biologics, patients on biologics tapering with flare-up had a significantly longer time interval between disease onset and initiation of cDMARDs (biologics tapering without flare-up: 0.27 (0.11–0.73) years; biologics tapering with flare-up: 1.14 (0.39–2.02) years; ever withdrawing biologics: 0.26 (0.18–0.42) years, *P* = 0.0104).

**Conclusion:** Juvenile ERA patients with polyarticular involvement had a higher risk of developing cDMARDs refractory and progressing to biologics use. Patients with a long time interval between disease onset and initiation of cDMARDs were prone to experience flare-up during tapering of biologics.

## Introduction

Juvenile spondyloarthritis (SpA) is a distinct entity of chronic pediatric arthritis with characteristics of male predominance, strong association with human leucocyte antigen (HLA)-B27, and involvement of the entheses and axial bones (1). Currently, there are seven subtypes of juvenile idiopathic arthritis (JIA), which are classified by the International League of Association for Rheumatology (ILAR) criteria (2). However, juvenile SpA was not one of the seven subtypes, and most juvenile SpA was categorized as enthesitis-related arthritis (ERA) according to the ILAR criteria (1).

Among the seven subtypes of JIA classified by ILAR, ERA is the most common in a large part of eastern and southern Asia, accounting for up to 30% of JIA cases (3, 4). In contrast, oligoarthritis is the most frequent subtype in North American JIA cohorts, while ERA only accounts for 10% of all JIA cases (5). Compared with other subtypes of JIA, children with ERA are prone to have higher disease activity and pain severity (6). However, possibly because of the relatively low prevalence of ERA in Western countries, limited literature has focused on the outcome and treatment response as well as the medication withdrawal rate in ERA patients (7–9).

Timely diagnosis and treatment of JIA with conventional disease-modifying anti-rheumatic drugs (cDMARDs) as well as biologics has dramatically changed the prognosis in the past two decades. Biologics, such as tumor necrosis factor inhibitors (TNFis), interleukin-6 antagonists, and T cell activation inhibitors, can be used in patients with active JIA refractory to cDMARDs. A large proportion of JIA patients have gained inactive disease status or even remission on medication. However, with the economic burden and other potential costs for patients, families, and society, as well as safety concerns regarding the long-term use of cDMARDs and/or biologics (10), important questions have arisen on how and when physicians can taper and/or withdraw medications. Another serious issue of post-withdrawal recurrence should also be emphasized. Studies on JIA treatment tapering and/or withdrawal varied in many aspects, such as enrolled population, medication studied and tapering protocol and outcome assessed (11). We aim to evaluate the proportion and characteristics of ERA patients in whom medications can be tapered in daily practice and to analyze the factors associated with flare-ups during medication tapering.

## Materials and Methods

We retrospectively reviewed the medical records of patients under 16 years old with a diagnosis of ERA from April 2001 to March 2020 at one tertiary medical center in Taiwan. The diagnosis of ERA was based on validated criteria defined by the ILAR (2). Patients were divided into two subgroups according to treatment: cDMARDs only and cDMARDs plus biologics. We retrieved demographic variables, laboratory parameters of inflammation, antinuclear antibody (ANA) positivity (titer > or = 1:80), HLA-B27 positivity, number of active joints at initiation of medication, presence of uveitis, presence of enthesitis, presence of axial involvement, type of cDMARDs administration, type of biologics use, time interval between disease onset and the start of cDMARDs, time interval between disease onset and the initiation of biologic therapy, time to achieve clinical inactive disease once biologic agent was started, time interval between clinical inactive disease and the initiation of biologics tapering, and medication withdrawal rate as well as post-withdrawal recurrence rate.

### Definition of Clinical Inactive Disease, Clinical Remission, Flare-Up, and Recurrence

We used Wallace criteria (12) to define clinical inactive disease, which included (1) no joints with active arthritis, (2) absence of systemic manifestations (fever, rash, serositis, splenomegaly or generalized lymphadenopathy resulting from JIA), (3) no active uveitis, (4) normal ESR or CRP (if both are tested, both must be within normal limits), and (5) physician's global assessment of disease activity indicating no disease activity.

Flare-up was defined as loss of at least two items of the Wallace criteria as well as when the attending physician intensified treatment because of elevated disease activity.

Clinical remission on medication was defined as clinical inactive disease for a minimum of 6 continuous months. Recurrence was defined as disease flare-up after clinical remission and discontinuation of cDMARDs and biologics for at least 2 months.

### Biologics Tapering Strategy

In our institution, most physicians reached a consensus to carefully extend the administration interval of biologics in patients with inactive disease, though there was no prespecified protocol. Tapering of cDMARDs was initiated before biologics. During tapering, concomitant administration of non-steroidal anti-inflammatory drugs (NSAIDs) was allowed. The decision of when to start tapering or the schedule of tapering biologics was left to the treating physician. The minimal follow-up period was 6 months after medication withdrawal.

### Statistical Analysis

Laboratory data are presented as the median (interquartile range, IQR). Continuous data were compared using the Kruskal-Wallis test. We compared categorical variables and proportions by using the chi-square test. Survival analysis was calculated by the Kaplan–Meier method. A threshold of *P* < 0.05 was used for statistical significance. Statistical analyses were conducted with R software (version 3.6.0).

## Results

### Patient Characteristics

There were 75 patients enrolled in this retrospective study. The demographic data and clinical characteristics of all patients and the two subgroups are summarized in Table 1. Among all patients, 19 (25.3%) patients took cDMARDs only, and 56 (74.7%) of them took cDMARDs plus biologics for disease control. There were 62 (82.7%) boys among all patients. The median onset age was 10.28 (IQR: 8.24–12.05) years old. The percentage of male patients and disease onset age showed no significant difference between the two subgroups.

**Table 1:** Characteristics of ERA patients with cDMARDs only and cDMARDs plus biologics.

	**Total (*N* = 75)**	**cDMARDs only (*N* = 19)**	**cDMARDs plus biologics (*N* = 56)**	***P*-value**
Onset age, years old	10.28 (8.24–12.05)	10.60 (8.28–12.20)	10.27 (8.24–12.04)	0.9127
Male sex	62/75 (82.7%)	14/19 (73.7%)	48/56 (85.7%)	0.2944
Poly-articular Oligo-articular	29/75 (38.7%) 46/75 (61.3%)	1/19 (5.3%) 18/19 (94.7%)	30/56 (53.6%) 26/56 (46.4%)	**0.0001**
ANA positivity	18/75 (24.0%)	0/19 (0%)	18/56 (32.1%a)	**0.0038**
HLA B27 positivity	75/75 (100.0%)	19/19 (100%)	56/56 (100%)	1.0
Uveitis	9/75 (12.0%)	1/19 (5.3%)	8/56 (14.3%)	0.4337
Enthesitis	20/75 (26.7%)	5/19 (26.3%)	15/56 (26.8%)	1.0
Axial involvement	28/75 (37.3%)	7/19 (36.8%)	21/56 (37.5%)	1.0
Time to cDMARDs, years	0.40 (0.20–1.24)	0.33 (0.11–0.53)	0.48 (0.23–1.39)	0.10006
MTX	54/75 (72.0%)	8/19 (42.1%)	46/56 (82.1%)	**0.0021**
SAL	31/75 (41.3%)	15/19 (78.9%)	16/56 (28.6%)	**0.0003**
AZA	21/75 (28.0%)	7/19 (36.8%)	14/56 (25.0%)	0.3796
HCQ	9/75 (12.0%)	5/19 (26.3%)	4/56 (7.1%)	**0.0406**
PEN	3/75 (4.0%)	1/19 (5.3%)	2/56 (3.6%)	1.0
CsA	3/75 (4.0%)	0/19 (0%)	3/56 (5.4%)	0.5667
Follow-up period, years	6.20 (2.91–9.56)	3.01 (1.04–5.30)	6.87 (4.59–11.35)	**0.00025**

*Data shown are median (IQR) or number (%) of patients as appropriate*.

*ERA, enthesitis-related arthritis; cDMARDs, conventional disease-modifying antirheumatic drugs; ANA, antinuclear antibody; HLA, human leucocyte antigen; MTX, methotrexate; SAL, sulfasalazine; AZA, azathioprine; HCQ, hydroxychloroquine; PEN, mesalazine; CsA, cyclosporine*.

*Values in the bold indicate p values less than 0.05*.

Overall, polyarticular involvement was noted in 29 (38.7%) patients, and it occurred more frequently in the cDMARDs plus biologics subgroup (cDMARDs only, 5.3%; cDMARDs plus biologics, 53.6%; *P* = 0.0001). ANA positivity was observed in 18 (24.0%) patients, and it occurred more frequently in the cDMARDs plus biologics subgroup (cDMARDs only, 0%; cDMARDs plus biologic agents, 32.1%; *P* = 0.0038). Nine (12.0%) patients had associated uveitis, and the incidence of uveitis showed no significant difference between the two subgroups. (*P* = 0.4337) Twenty (26.7%) patients had enthesitis, while 28 (37.3%) patients presented with axial involvement. The incidence of enthesitis and axial involvement showed no significant difference between the two subgroups.

The median time interval between disease onset and the start of cDMARDs was 0.40 (IQR: 0.20–1.24) years, and there was no significant difference between these two subgroups. The two most commonly used cDMARDs were methotrexate and sulfasalazine, which were prescribed to 72.0% and 41.3% of the patients, respectively. Methotrexate (82.1%) was the most commonly prescribed cDMARD in the cDMARDs plus biologics subgroup, while sulfasalazine (78.9%) was the most frequently used cDMARD in the cDMARDs only subgroup.

### Medication Withdrawal Rate and Post-withdraw Recurrence Rate

The overall medication withdrawal rate was 34.7%, and it occurred more frequently in patients with cDMARDs only (cDMARDs only, 84.2%; cDMARDs plus biologics, 17.9%; *P* < 0.001). Post-withdrawal recurrence occurred in 10 (38.5%) patients, and half of them occurred within 1 year after discontinuation of all medication. The post-withdrawal recurrence rate showed no significant difference between these two subgroups (cDMARDs only, 31.3%; cDMARDs plus biologics, 50.0%; *P* = 1.0) (see Table 2).

**Table 2:** Medication withdrawal rate and post-withdrawal recurrence rate in ERA patients with cDMARDs only and cDMARDs plus biologics.

	**Total *N* = 75**	**cDMARDs only *N* = 19**	**cDMARDs plus biologics *N* = 56**	***P*-value**
Medication withdrawal rate	26/75 (34.7%)	16/19 (84.2%)	10/56 (17.9%)	** <0.00001**
Recurrence^a^ rate	10/26 (38.5%)	5/16 (31.3%)	5/10 (50.0%)	0.425
Recurrence within 1 year	5/10 (50.0%)	2/5 (40.0%)	3/5 (60.0%)	1.0
Recurrence after 1 year	5/10 (50.0%)	3/5 (60.0%)	2/5 (40.0%)	1.0

*Data shown are number (%) of patients as appropriate*.

*ERA, enthesitis-related arthritis; cDMARDs, conventional disease-modifying antirheumatic drugs*.

a *Recurrence was defined as disease flare-up after clinical remission and discontinuation of cDMARDs and biologic agents for at least 2 months*.

*Values in the bold indicate p values less than 0.05*.

### Factors Associated With Flare-Up During Biologics Tapering

**Figure 1:**
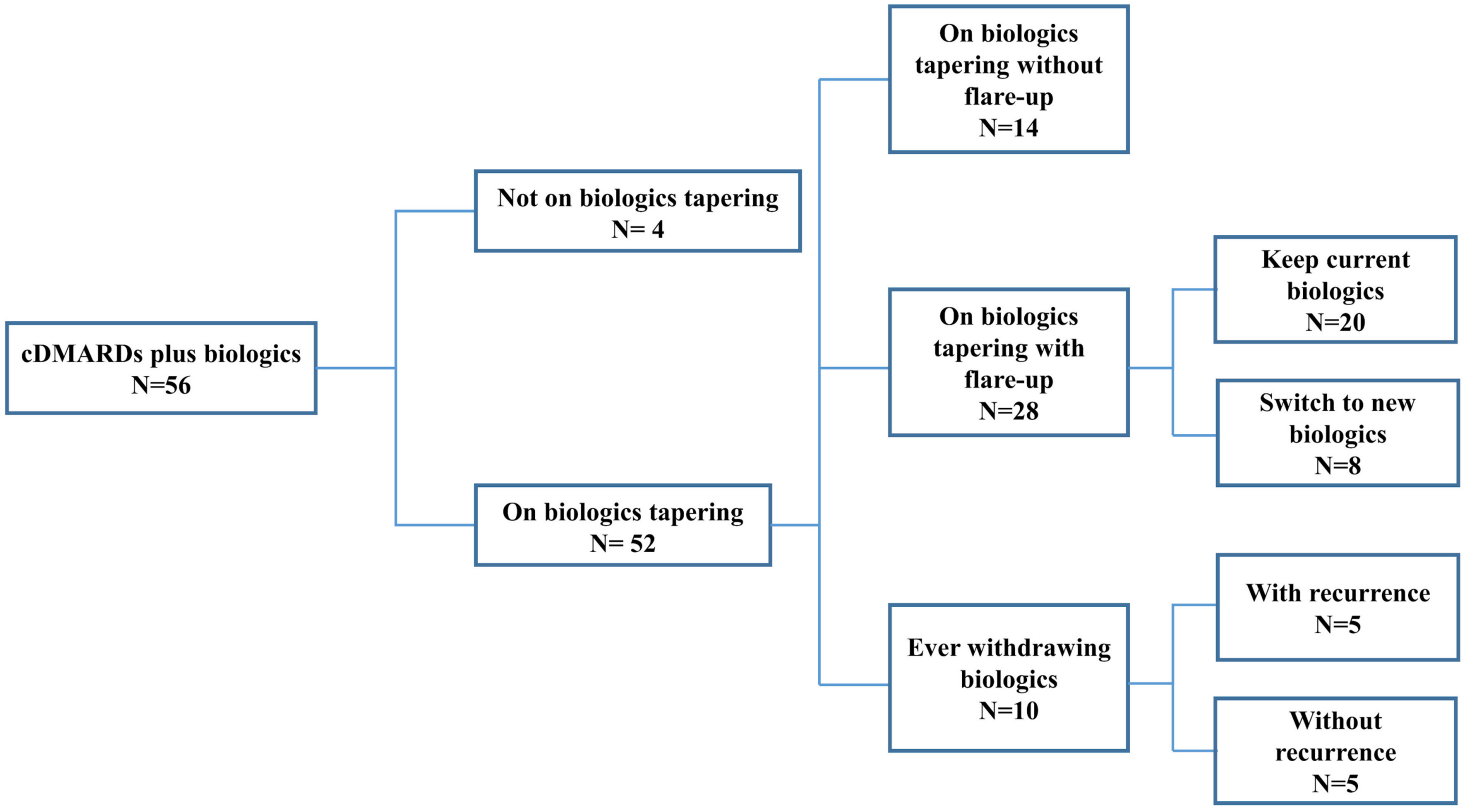
ERA patients stratified by biologics tapering status. ERA, enthesitis-related arthritis; cDMARDs, conventional disease-modifying antirheumatic drugs.

**Figure 2:**
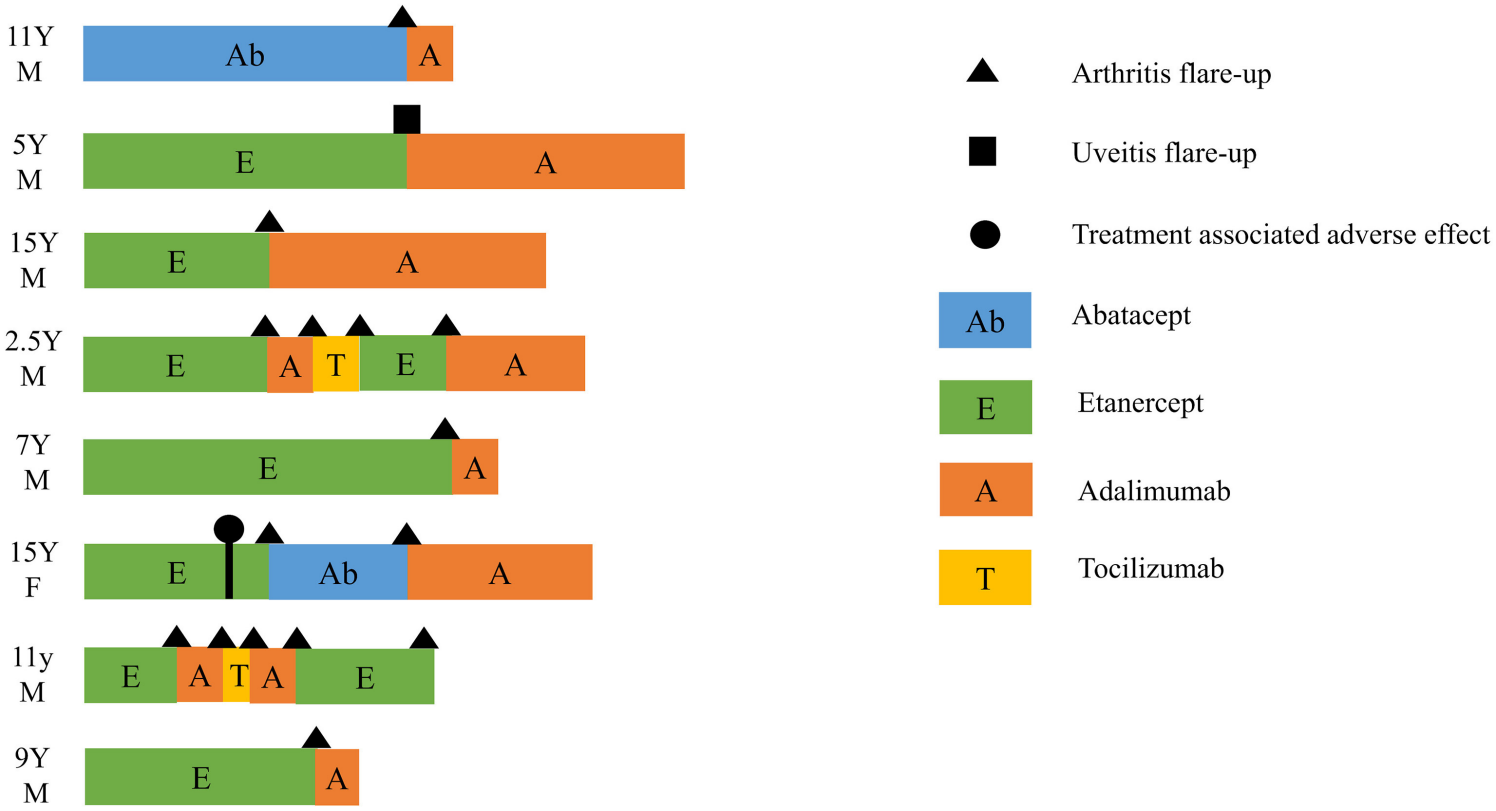
Switch between biologics in eight patients who had flare-up during biologics tapering. Triangle, flare-up with presentation of arthritis; square, flare-up with presentation of uveitis; circle, treatment-associated adverse effect (pulmonary tuberculosis infection); Ab, abatacept; E, etanercept; A, adalimumab; T, tocilizumab; Y, years old; M, male; F, female.

To further investigate clinical predictors of successful tapering and then discontinuation of biological agents, we categorized the patients with cDMARDs plus biologics into four subgroups based on whether they experienced flare-up during tapering of biologics: not on biologics tapering, on biologics tapering with flare-up, on biologics tapering without flare-up, and ever withdrawing biologics. There were 4 patients who had not been on biologics tapering, 14 on biologics tapering without flare-up, 28 on biologics tapering with flare-up, and 10 ever withdrawing biologics. Half of the ever withdrawing biologics subgroup experienced recurrence during the follow-up period (see Figure 1). Among all patients with cDMARDs plus biologics, 39 (69.6%) patients used etanercept as first-line biologic, 16 (28.6%) patients used adalimumab as first-line biologic, and only one patient (1.8%) used abatacept as first-line biologic. In the biologics tapering with flare-up subgroup, eight patients switched to a second-line biological agent for better disease control. The detailed biologic switches of the eight patients is illustrated in Figure 2. Seven (87.5%) of them who had biologic switches were male. Seven (87.5%) patients received etanercept as first-line biologic, and one (12.5%) patient took abatacept as first-line biologic. Abatacept was used as first-line treatment instead of TNFi in this patient due to a history of severe skin eruption after TNFi injection. (He previously received a single etanercept injection, which precipitated the skin eruption.) The most commonly used biologic as a second-line treatment during flare-ups was adalimumab (87.5%). One patients used abatacept as second-line treatment after etanercept failed and due to an etanercept-related adverse effect (pulmonary tuberculosis). More than one biologic switch occurred in three patients, and two of them used tocilizumab as a third-line treatment. Flare-up with presentation of active arthritis was noted in seven patients, and uveitis was noted in one patient. Among 56 patients who received biologics, only one patient was found to have pulmonary tuberculosis after 2.5 years of etanercept (see Figure 2).

Among the three subgroups (on biologics tapering without flare-up, on biologics tapering with flare-up, ever withdrawing biologics), the disease onset age, percentage of male patients, poly-articular involvement, ANA positivity, presence of uveitis, presence of enthesitis, presence of axial involvement, laboratory parameters of inflammation, and type of administered cDMARDs and biologics showed no significant difference. Patients on biologics tapering with flare-up had a significantly longer time interval between disease onset and initiation of cDMARDs (on biologics tapering without flare-up: 0.27 (0.11–0.73) years; on biologics tapering with flare-up:1.14 (0.39–2.02) years; ever withdrawing biologics: 0.26 (0.18–0.42) years, *P* = 0.0104). Patients on biologics tapering with flare-up also seemed to take a longer time to achieve clinical inactive disease once the biological agent was started though with only trend significance (on biologics tapering without flare-up:0.35 (0.33–0.44) years; on biologics tapering with flare-up:0.38 (0.21–0.52) years; ever withdrawing biologics: 0.27 (0.16–0.30) years, *P* = 0.0948). The median time interval to biologics tapering after achieving clinical inactive disease was 0.57 (0.30–0.84) years, and it showed no significant difference among the three subgroups (see Table 3).

**Table 3:** Demographics and clinical manifestations in ERA patients stratified by biologics tapering.

	**On biologics tapering without flare-up (*N* = 14)**	**On biologics tapering with flare-up (*N* = 28)**	**Ever withdrawing biologics (*N* = 10)**	***P*-value**
Onset age, years old	10.41 (8.43–11.91)	10.19 (6.85–12.25)	10.27 (9.03–10.78)	0.9147
Male	12/14 (85.7%)	24/28 (85.7%)	10/10 (100.0%)	0.6114
Polyarticular oligoarticular	7/14 (50.0%) 7/14 (50.0%)	14/28 (50.0%) 14/28 (50.0%)	6/10 (60.0%) 4/10 (40.0%)	0.8686
ANA positivity	4/14 (28.6%)	9/28 (32.1%)	3/10 (30.0%)	1.0
Uveitis	3/14 (21.4%)	4/28 (14.3%)	1/10 (10.0%)	0.7697
Enthesitis	4/14 (28.6%)	9/28 (32.1%)	2/10 (20.0%)	0.9188
Axial involvement	2/14 (14.3%)	11/28 (39.3%)	6/10 (60.0%)	0.0743
CRP mg/dl	0.98 (0.33–3.30)	2.05 (1.14–2.93)	1.91 (0.69–3.16)	0.5828
ESR mm/hr	31.50 (17.75–44.25)	37.00 (27.00–62.00)	29.00 (14.00–53.00)	0.4141
**Initial biologics**				
Etanercept	8/14 (57.1%)	21/28 (75.0%)	8/10 (80.0%)	
Adalimumab	6/14 (42.9%)	6/28 (21.4%)	2/10 (20.0%)	
Abatacept	0/14 (0%)	1/28 (3.6%)	0/10 (0%)	0.4975
Time to cDMARDs, years	0.27 (0.11–0.73)	1.14 (0.39–2.02)	0.26 (0.18–0.42)	**0.0104**
MTX	11/14 (78.6%)	23/28 (82.1%)	9/10 (90.0%)	0.8906
SAL	3/14 (21.4%)	9/28 (32.1%)	2/10 (20.0%)	0.7637
AZA	3/14 (21.4%)	8/28 (28.6%)	2/10 (20.0%)	0.8346
HCQ	2/14 (14.3%)	2/28 (7.1%)	0/10 (0%)	0.6351
PEN	1/14 (7.1%)	0/28 (0%)	1/10 (10%)	0.2081
CsA	1/14 (7.1%)	1/28 (3.6%)	1/10 (10%)	0.7605
Time to biologics, years	0.88 (0.58–2.01)	1.39 (0.54–4.72)	0.92 (0.40–2.30)	0.5136
Time to achieve clinical inactive disease once biological agent was started, years	0.35 (0.33–0.44)	0.38 (0.21–0.52)	0.27 (0.16–0.30)	0.0948
**Concomitant treatment while achieving clinical inactive disease**				
cDMARDs	8/14 (57.1%)	12/28 (42.9%)	5/10 (50.0%)	0.6597
NSAIDs	5/14 (35.7%)	7/28 (25.0%)	0/10 (0%)	0.1062
Corticosteroid	0/14 (0%)	2/28 (7.1%)	0/10 (0%)	0.7043
Time to biologic tapering after clinical inactive disease was achieved, years	0.55 (0.24–1.26)	0.73 (0.34–1.28)	0.49 (0.37–0.57)	0.7418
**Concomitant treatment while initiating biologics tapering**				
cDMARDs	2/14 (14.3%)	6/28 (21.4%)	2/10 (20.0%)	0.8982
NSAIDs	1/14 (7.1%)	5/28 (17.9%)	1/10 (10.0%)	0.8607
Corticosteroid	0/14 (0%)	1/28 (3.6%)	0/10 (0%)	1.0
Follow-up period, years	5.34 (3.05–7.43)	9.71 (6.32–13.35)	6.85 (6.21–7.90)	**0.0225**

*Data shown are median (IQR) or number (%) of patients as appropriate*.

*ERA, enthesitis-related arthritis; cDMARDs, conventional disease-modifying antirheumatic drugs; ANA, antinuclear antibody; CRP, C-reactive protein; ESR, erythrocyte sedimentation rate; MTX, methotrexate; SAL, sulfasalazine; AZA, azathioprine; HCQ, hydroxychloroquine; PEN, mesalazine; CsA, cyclosporine; NSAIDs, non-steroidal anti-inflammatory drugs*.

*Values in the bold indicate p values less than 0.05*.

*The value with underline indicates p values between 0.05 and 0.1.(trend)*.

When they achieved clinical inactive disease, 25 (48.1%) patients were taking cDMARDs, 12 (23.1%) patients were taking NSAIDs, and NSAIDs were mostly used for pain relief only, not on a daily basis. Two (3.8%) patients were under corticosteroids, and the corticosteroid dose was 5 mg of prednisolone per day. When biologics tapering was initiated, 10 (19.2%) patients were taking cDMARDs, 7 (13.5%) patients were taking NSAIDs, and 1 (1.9%) patient was taking corticosteroids (see Table 3).

**Figure 3:**
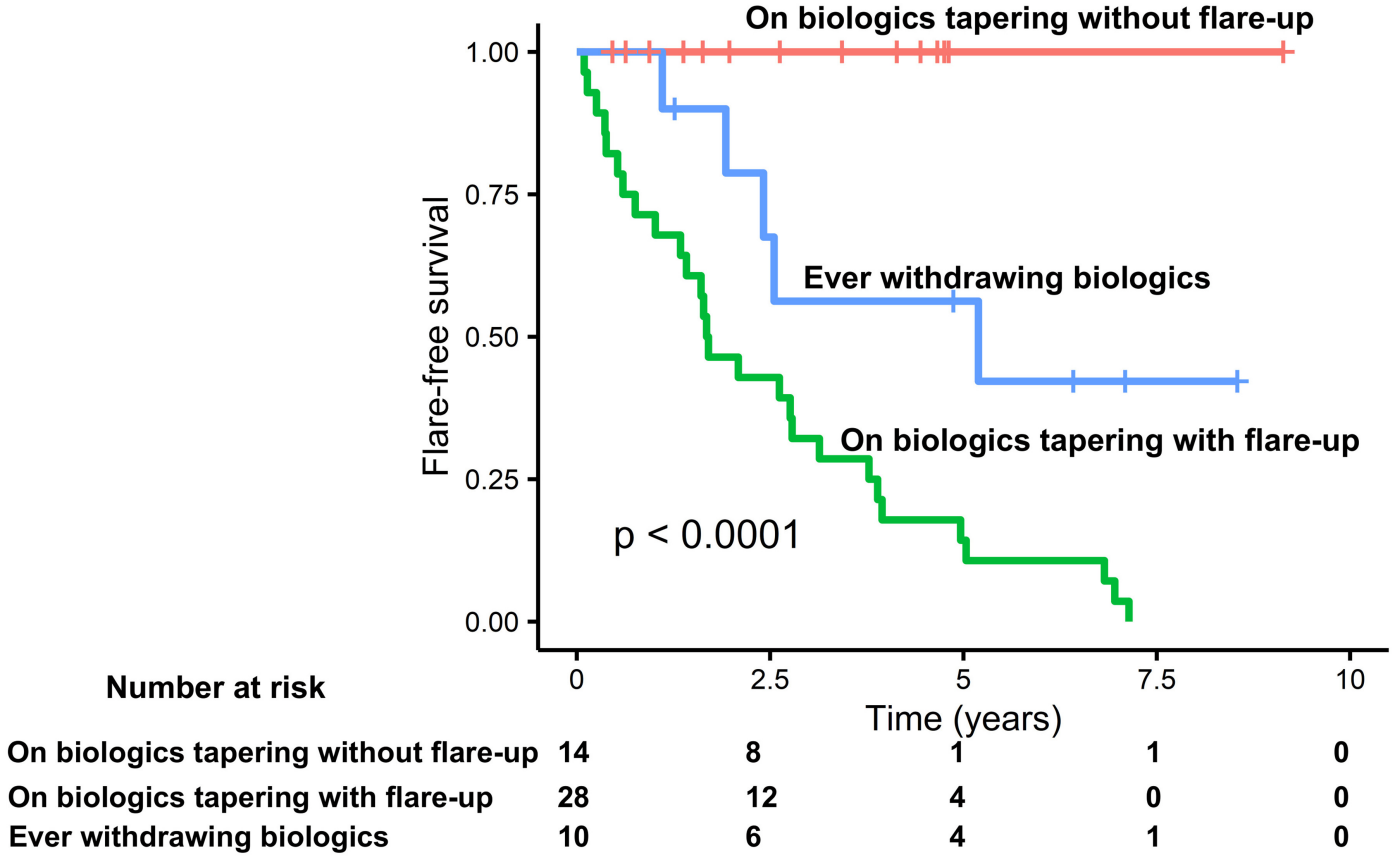
Kaplan–Meier plot of time to enthesitis related arthritis (ERA) disease flare-up. Flare-up was defined as loss of at least two items of the Wallace criteria as well as when the attending physician intensified treatment because of elevated disease activity. Time to flare was defined as the time from the date initiating biologics tapering to the date of the first flare visit. Patients without flare-up were censored at the date of the latest visit.

Kaplan–Meier survival analysis demonstrated that the flare-free survival rate was significantly higher in the biologics tapering without flare-up group than in the biologics tapering with flare-up group and in the ever withdrawing biologics group (*P* < 0.0001). The median time to flare-up was 1.69 years in the biologics tapering with flare-up group versus 5.19 years in the ever withdrawing biologics group (see Figure 3).

## Discussion

With more JIA patients achieving constant remission under cDMARDs and/or biologics, tapering or even withdrawing medications has been taken into account by the patients as well as the attending physicians. However, in a multicenter prospective observational study conducted by Otten et al. a sustained remission status could not be achieved in 22 pediatric patients with ERA, and none of them discontinued TNFi successfully. The study may not fully reflect the real-world data of biologics use in ERA patients not only because of limited case numbers but also because of the relatively high drop-out rate (up to two-thirds of them were lost to follow-up after 2.25 years) (7). Herein, we evaluated the proportion and characteristics of 75 ERA patients in whom medications can be tapered in daily practice and analyzed the factors associated with flare-ups during medication tapering.

In the present study, the overall medication withdrawal rate was 34.7% within the median follow-up period of 6.2 years. The withdrawal rate was significantly higher in patients with cDMARDs only (84.2%), and this may be correlated with variable disease severity (active joint number at initiation of medications) between cDMARDs only and cDMARDs plus biologic subgroups. The incidence of polyarticular involvement was 10-fold higher in patients receiving cDMARDs plus biologics than in patients receiving cDMARDs only.

The majority of our ERA patients had oligo-articular involvement (61.3%); however, the percentage was significantly lower compared to previous cohort studies (74–90%) (13–15). This discrepancy might relate to different ethnicities or study design. In previous literature, oligoarthritis or polyarthritis was defined by the active joint count at disease onset or diagnosis; however, we defined oligo-articular or poly-articular involvement by the active joint count at initiation of cDMARDs. Referral bias could also contribute to the difference in patient characteristics. Our institution, as a tertiary referral center, often cares for patients with higher disease severity, who often have polyarthritis, high inflammatory biomarkers, or are refractory to conventional treatment. Therefore, patients with poly-articular involvement may be overrepresented in our cohort study.

We also found that patients with a longer time interval between disease onset and initiation of cDMARDs had a higher risk of flare-ups during tapering of biologics. This finding demonstrated that earlier initiation of cDMARDs increased the likelihood of successful treatment withdrawal, possibly owing to the prevention of chronic and irreversible joint damage. Our report also disclosed that patients who had a shorter time to achieve clinical inactive disease once biological agents were started seemed to have a lower chance of experiencing flare-up during biologics tapering, though only with trend significance, which may be related to the limited number of cases.

When patients experienced flare-ups during biologics tapering, the treating physicians would pause the tapering attempt, escalate treatment to previous step, or even shift to alternative biologics in order to keep disease activity at inactive status. Further attempts at tapering might be considered with great prudence either by the treating physicians or patients themselves (16). Thus, patients with flare-ups during biologics tapering might receive a long period of follow-up with multiple stops and starts. We only analyzed the first tapering attempt for each patient, but the follow-up period was extended since the patients were still in need of active treatment. By contrast, some patients who completed tapering achieved total withdrawal of biologics, and were lost to follow-up, since these patients had no more active medical needs. This explains the shorter period of clinical follow-up for the ever withdrawing biologics group.

ANA positivity was noted more frequently in patients under biologics, and it corresponded to previous studies disclosing that TNFi treatment was associated with the development of ANA (17). It remains controversial whether ANA can be a biomarker predicting poor response to biologic treatment. Some studies reported that ANA and anti-ds DNA were associated with poor outcome to biological agents in patients with rheumatoid arthritis (18, 19). However, ANA was not associated with flare-ups in ERA patients receiving biologics in our study, and this finding was compatible with the study conducted by Simonini et al. in patients with JIA (20).

The most frequently used cDMARD in patients receiving cDMARDs only is sulfasalazine. This result was compatible with the therapeutic recommendation advised by the American College of Rheumatology in 2011 (21). Although methotrexate is known to be less effective in patients with ERA (21), JIA patients in Taiwan have to take methotrexate first for at least 3 months before applying biologics regardless of the subtypes to which these patients belong. If methotrexate usage of 3 months proved to be ineffective or intolerable to patients, patients could apply for biologics covered by national health insurance instead of at their own expense. This explained why patients under biologics took methotrexate more frequently than others. Twenty-one (28%) and nine (12%) patients took azathioprine and hydroxychloroquine, respectively. Though less commonly used in ERA patients, azathioprine has been reported as a beneficial alternative for SpA (22, 23) and JIA associated uveitis (24). Hydroxychloroquine, though less commonly used in patients with SpA, showed greater efficacy while in combination with methotrexate and sulfasalazine compared with sulfasalazine alone (25).

TNFis were the most commonly used first-line biologics in our cohort study, comprising 98.2% subjects (etanercept: 69.6%, adalimumab: 28.6%), and this result was compatible with the therapeutic recommendation advised by the American College of Rheumatology in 2019 for JIA children with active enthesitis or sacroiliitis (26). Switching between biologics occurred in 8 patients (six to adalimumab after failing etanercept, one to adalimumab after failing abatacept, and one to abatacept after failing etanercept). Three (37.5%) patients discontinued second-line biologics due to ineffectiveness and switched to third-line biologics. The therapeutic options of biologics were limited, because only four kinds of biologics were reimbursed by the national health insurance for JIA patients in Taiwan (etanercept, adalimumab, abatacept, and tocilizumab). Treatment of JIA patients whose disease failed to respond to TNFi or who could not tolerate TNFi was challenging. When the first TNFi failed to show efficacy, physicians might choose another TNFi, abatacept, or tocilizumab as alternatives. Although there was no strong evidence about the effectiveness of biologics switching, it is still attempted since only limited treatment options were available.

Scant literature focused on the efficacy of abatacept and tocilizumab in ERA children; however, both biologics failed to demonstrated major clinical improvement in adult patients with spondyloarthritis (27). Recent research interest has concentrated on the IL-23/IL-17 axis. Blockade of IL-23 or IL-17 worked well in adult patients with ankylosing spondylitis (28, 29). Trials of secukinumab (an anti-IL-17A monoclonal antibody) have been launched in children with ERA (NCT03031782, NCT03769168).

Etanercept was the most commonly used first-line biologics in our cohort, up to 69.6%. One patient was infected with pulmonary tuberculosis after 2.5 years of etanercept. TNFi has already been proven to increase the risk of severe infection, especially tuberculosis (30). Therefore, latent tuberculosis infection screening before TNFi is warranted, especially in countries with a high tuberculosis burden (31).

There were several limitations in our cohort study. With its retrospective nature, a risk of missing data or incorrect documentation may exist. Second, it is difficult to derive definitive conclusions from this single-center experience with limited case numbers. However, few previous investigations exist on juvenile ERA patients with long follow-up periods, and the current pilot study provides new insights in this subgroup. Further research with multiple centers or nationwide databases is warranted. Finally, there was no strict medication tapering protocol in this study. Attending physicians mostly tapered cDMARDs first and then extended intervals between doses of biologics. The speed of tapering was based on physicians' judgment. Although this tapering strategy was more practical in daily care, a rigorous study design with a fixed tapering protocol (whether dose reduction or dose interval extension) is still needed to identify factors associated with successful biologic tapering.

Thus far, there are few studies based on daily routine care that focus on the medication withdrawal rate in children with ERA. We found that approximately half of the biologics users experienced flare-ups during tapering, and half of those who halted medication successfully had post-withdrawal recurrence. A longer time interval between disease onset and initiation of cDMARDs was associated with flare-up during medication tapering. Therefore, early intervention with cDMARDs may decrease the incidence of flare-ups during tapering and further increase the medication withdrawal rate in ERA patients.

## Data Availability Statement

The raw data supporting the conclusions of this article will be made available by the authors, without undue reservation.

## Ethics Statement

The studies involving human participants were reviewed and approved by The Ethics Committee of National Taiwan University Hospital (202010068RIND). Written informed consent from the participants' legal guardian/next of kin was not required to participate in this study in accordance with the national legislation and the institutional requirements.

## Author Contributions

C-HL led the overall study, contributed to the data collection and interpretation, and wrote the manuscript. B-LC contributed to the data interpretation and manuscript editing. Y-HY contributed to the study design and manuscript editing. All authors read, contributed to the research design, and approved the final manuscript.

## Conflict of Interest

The authors declare that the research was conducted in the absence of any commercial or financial relationships that could be construed as a potential conflict of interest.
